# LGE in pulmonary hypertension predicts clinical events

**DOI:** 10.1186/1532-429X-13-S1-P297

**Published:** 2011-02-02

**Authors:** Siva Soma, Srinivas Murali, Raymond Benza, June A Yamrozik, Ronald B Williams, Mark Doyle, Diane Vido, Robert WW Biederman, Vikas Rathi

**Affiliations:** 1 Allegheny General Hospital, Pittsburgh, PA, USA; 2 Bon Secours Heart and Vascular Institute, Richmond, VA, USA

## Introduction

### Introduction

Right ventricular (RV) function predicts prognosis in pulmonary hypertension (PH) patients (pts) and Right Ventricular failure (RV). Prior studies evaluating of 3D RV ejection fraction (EF) have yielded inconsistent prognostic information. Here we explore the prognostic value of contrast induced CMR in PH patients with RV dysfunction.

## Purpose

### Hypothesis

We hypothesize that myocardial Late Gadolinium Enhancement (LGE) which a marker for myocardial fibrosis when present in RV or RV insertion points (RVIP) is a predictor of poor prognosis in PH pts.

## Methods

A retrospective chart review of PH pts (n=21) who underwent clinically indicated CMR were analyzed. Demographic data showed mean age 61 yrs; 28% male; 43 % WHO group I, 29% group II, 14% group IV,14% group V. RV volumetric data were indexed to BSA, and along with RVIP LGE information were correlated with major adverse clinical events (MACE) such as hospitalization, death and referral/need for lung transplantation.

## Results

LGE was positive (+) in 14 pts (66%) and (-) in the remaining 7 pts (33%). Compared to LGE (- ) pts, the LGE (+) pts had higher RVEDVI, RVESVI and lower RVEF (p<0.05). However, only LGE in combination with 3D RVEF predicted MACE. Specifically, LGE (+) pts had 9 MCE compared to 1 in the LGE (-) pts. The median RVEF value was 45%. The event rate was 89% in pts with LGE (+) and RVEF<45 compared to 20% in LGE (-) and RVEF <45%. Group comparisons were done using the Fisher’s exact test. The comparisons were not statistically significant due to small sample size.

## Conclusions

Late Gadolinium Enhancement may be the pathophysiologic hallmark in patients with PH patients as a direct reflection of the underlying RV failure due to progressive myocardial fibrosis. Late gadolinium enhancement in combination with RVEF provides important prognostic information that accurately predicts important adverse CV outcomes such as lung transplantation and death. Prospective studies are needed to confirm this observation.

